# Unlocking Short Read Sequencing for Metagenomics

**DOI:** 10.1371/journal.pone.0011840

**Published:** 2010-07-28

**Authors:** Sébastien Rodrigue, Arne C. Materna, Sonia C. Timberlake, Matthew C. Blackburn, Rex R. Malmstrom, Eric J. Alm, Sallie W. Chisholm

**Affiliations:** Department of Civil and Environmental Engineering, Massachusetts Institute of Technology, Cambridge, Massachussetts, United States of America; Plymouth Marine Laboratory, United Kingdom

## Abstract

**Background:**

Different high-throughput nucleic acid sequencing platforms are currently available but a trade-off currently exists between the cost and number of reads that can be generated versus the read length that can be achieved.

**Methodology/Principal Findings:**

We describe an experimental and computational pipeline yielding millions of reads that can exceed 200 bp with quality scores approaching that of traditional Sanger sequencing. The method combines an automatable gel-less library construction step with paired-end sequencing on a short-read instrument. With appropriately sized library inserts, mate-pair sequences can overlap, and we describe the SHERA software package that joins them to form a longer composite read.

**Conclusions/Significance:**

This strategy is broadly applicable to sequencing applications that benefit from low-cost high-throughput sequencing, but require longer read lengths. We demonstrate that our approach enables metagenomic analyses using the Illumina Genome Analyzer, with low error rates, and at a fraction of the cost of pyrosequencing.

## Introduction

New DNA sequencing technologies are dramatically changing the research landscape in biology by enabling experiments that were previously too expensive or time-consuming. Three platforms, the Roche-454 Genome Sequencer, the Illumina Genome Analyzer II and the Applied Bio-Systems SOLiD system, are widely used [1]. The latter two instruments produce a very large number of short reads, typically 25–75 nucleotides long, at a significantly lower cost per basepair (bp) compared to the Roche-454 pyrosequencing system. However, short reads create difficulties for some applications such as metagenomics and meta-transcriptomics, and therefore pyrosequencing remains the platform of choice. Efforts have been made to obtain longer sequences from short-read instruments, and further expand their range of applications [2], [3]. A clever strategy was recently developed by Hiatt and colleagues, but their approach requires many steps and imposes a library complexity bottleneck that can limit its utility for applications like metagenomics [2]. We sought to establish a more straightforward and broadly applicable method. Our approach builds on preparing libraries with tunable size distributions such that sequencing from both ends generates overlapping reads. The lower-quality ends of mated reads can then be overlapped to produce high-quality consensus sequence. We demonstrate our strategy using the Illumina GAII technology although the procedures we describe could be applied to other sequencing platforms that can generate paired end data.

## Results

**Figure 1:**
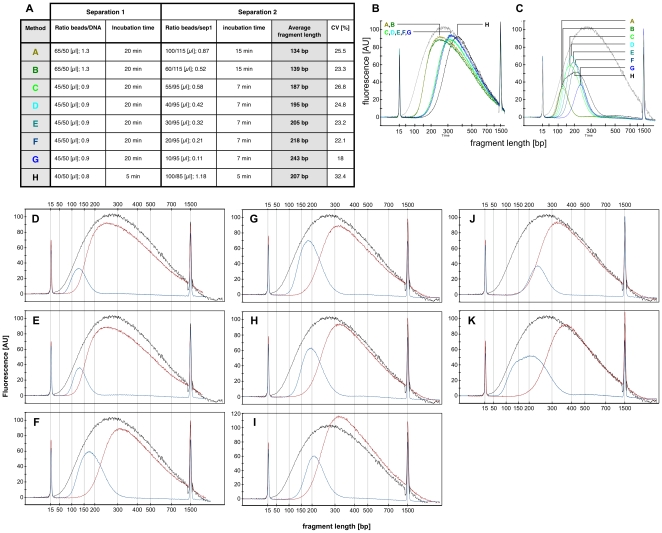
Size-dependent isolation of DNA fragments from sheared genomic DNA via dSPRI. AMPure XP SPRI beads bind DNA fragments in a size dependent manner according to the concentration of salts and polyethylene glycol (PEG) in the reaction [4]–[7], which can easily be changed by using different volume ratios of DNA to SPRI bead solutions. A two-step procedure is employed to isolate targeted DNA size fractions. Panels A to H present Bioanalyzer DNA-1000 assays showing the sheared genomic DNA used as starting material (black), the larger size DNA fragments discarded in separation 1 (red), and the size fraction purified and recovered after separation 2 (blue). Panel I is a table summarizing the conditions and results displayed in panels A to H. All Bioanalyzer DNA-1000 traces after separation 1 (panel J), and after separation 2 (panel K), are respectively displayed on a graph for the conditions presented in panels A to H. The conditions displayed in panel H were used to obtaine the Illumina composite reads discussed in the text. The wider DNA fragment size distribution from panel H allowed to better analyze the effects of shorter versus longer overlapping regions on consensus reads.

**Figure 2:**
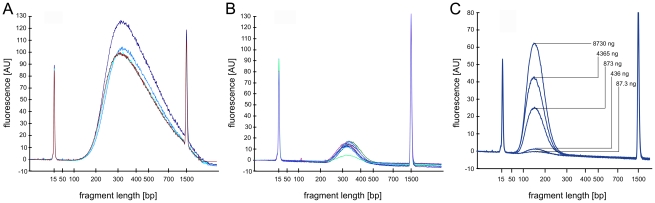
Reproducibility of double-SPRI. The panel shows DNA fragment size distributions as obtained by Bioanalyzer DNA-1000 assays. The curves represent the size fractions removed during the first separation step **A**) or recovered after the second separation **B**). The two size fractions were independently reproduced in 4 or 8 separation experiments. The curves in **B**) represent the libraries sequenced after dSPRI based size selection, adapter ligation and PCR enrichment. While concentrations (arbitrary fluorescence units) vary between reproduced libraries the range of removed or enriched DNA fragment sizes was highly reproducible. Panel **c**) shows the DNA fragment size distribution recovered after the second separation when using decreasing amounts sheared genomic DNA. dSPRI allows reliable size selection in a DNA concentration independent manner.

We developed a simple and automatable protocol to rapidly, efficiently and reproducibly isolate DNA fragments of a specific size distribution without the need for gel electrophoresisis. The method is similar to a recently published protocol [4] but allows adjusting the size distribution of the isolated DNA fragments as appropriate for the sequencing platform used. After ultrasonic DNA shearing, size-selection is carried out via double solid phase reversible immobilization (dSPRI) using carboxyl coated magnetic beads (Figure 1). DNA associates with these particles in a strictly size-dependent manner according to the concentration of polyethylene glycol and salts [4]–[7], which can be adjusted by changing the volume ratio of SPRI bead suspension to DNA solution (Figure 1). The size distribution of selectively bound DNA is concentration independent (Figure 2), which supports the preparation of sequencing libraries from dilute samples. In the first step of this procedure, DNA fragments larger than the desired library insert size are bound to magnetic beads, which are then discarded. The supernatant is saved, and mixed with an appropriate volume of fresh SPRI beads. This second incubation preferentially immobilizes DNA fragments of the targeted size range. Shorter DNA molecules, salts and other reagents are removed by wash steps, and the DNA fraction that remains bound to the magnetic SPRI beads can subsequently be recovered with virtually no loss [4]. The resulting DNA population reproducibly exhibits a relatively narrow size distribution (Figure 2), and can be used to prepare high-throughput sequencing libraries by ligating appropriate oligonucleotide adapters (Table 1).

**Table 1:** Oligonucleotides sequences.

Name	Purification	Sequence (5' to 3')
IGA-A-down	HPLC	B'B'B'B' AGA TCG GAA GAG CGT CGT GTA GGG AAA GAG TGT AC/3AmM/
IGA-A-up	HPLC	/5AmMC6/ACA CTC TTT CCC TAC ACG ACG CTC TTC CGA TCT BBBB
IGA-PE-B0-down	HPLC	/5AmMC6/CTC GGC ATT CCT GCT GAA CCG CTC TTC CGA TCT
IGA-PE-B0-up	HPLC	AGA TCG GAA GAG CGG TTC AGC AGG AAT GCC GAG /3AmM/
IGA-PCR-PE-F	PAGE	AAT GAT ACG GCG ACC ACC GAG ATC TAC ACT CTT TCC CTA CAC GAC GCT CTT CCG ATC T
IGA-PCR-PE-R	PAGE	CAA GCA GAA GAC GGC ATA CGA GAT CGG TCT CGG CAT TCC TGC TGA ACC GCT CTT CCG ATC T
QPCR-library quantification-F	Standard desalting	AAT GAT ACG GCG ACC ACC GA
QPCR-library quantification-R	Standard desalting	CAA GCA GAA GAC GGC ATA CGA

All oligonucleotides were purchased from IDT (www.idtdna.com).

5AmMC6: 5' Amino Modifier C6.

3AmM: 5' Amino Modifier.

B: Barcode base. The sequencing reads start with these bases. The barcodes used in this study were AAAC, ACCC, AGGC and ATTC.

B': Complementary barcode base when the two oligonucleotide from the same adapter are annealed.

**Figure 3:**
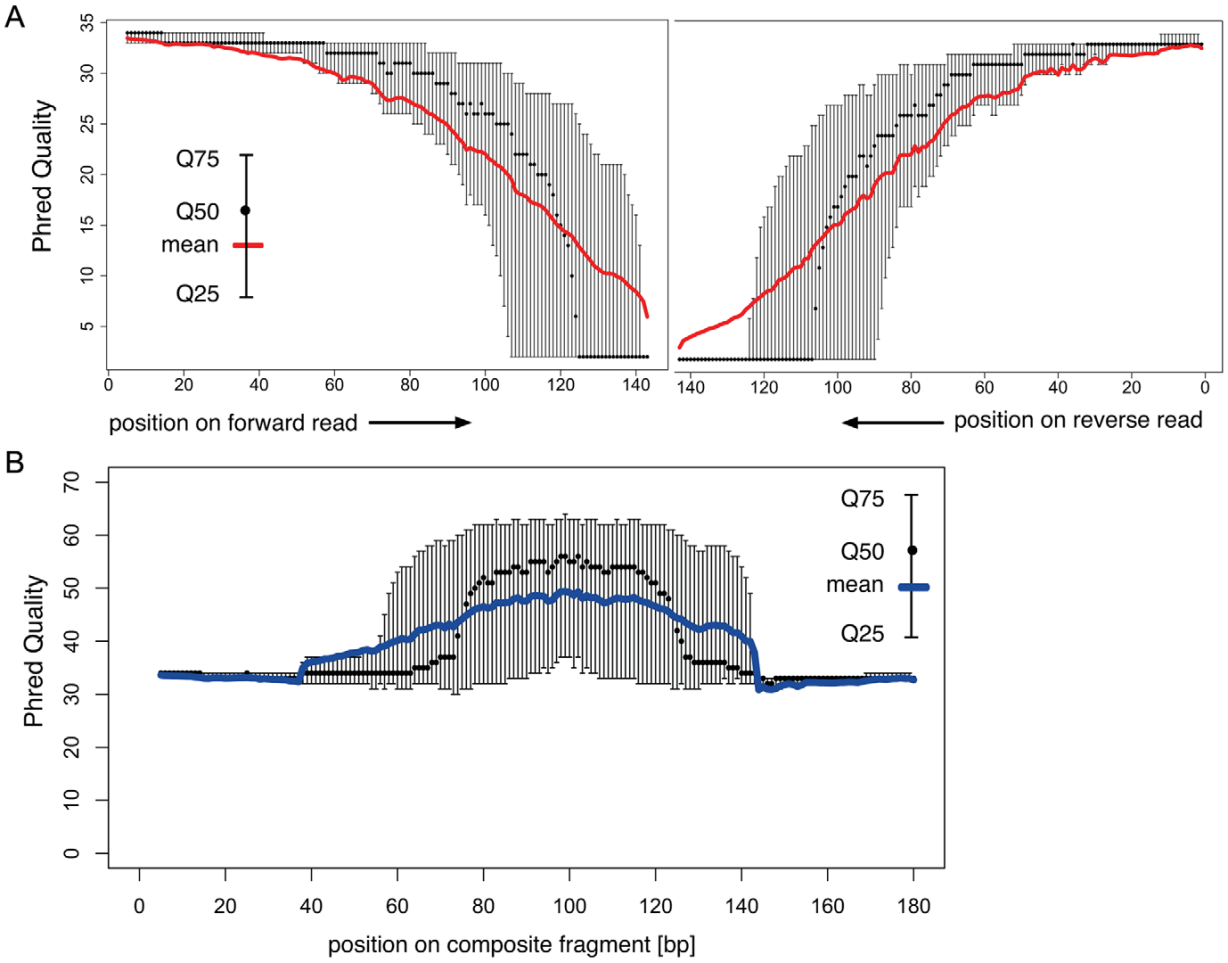
Quality of Illumina reads out to 143 bp. **A**) The mean Phred quality of single reads is shown in the solid red lines, with error bars displaying quartiles. Read quality is highly variable toward the reads' ends. Read quality as a function of base pair is worse on the second mate of the pair. **B**) The mean and quartiles of Phred quality by base for the average-length composite read.

**Figure 4:**
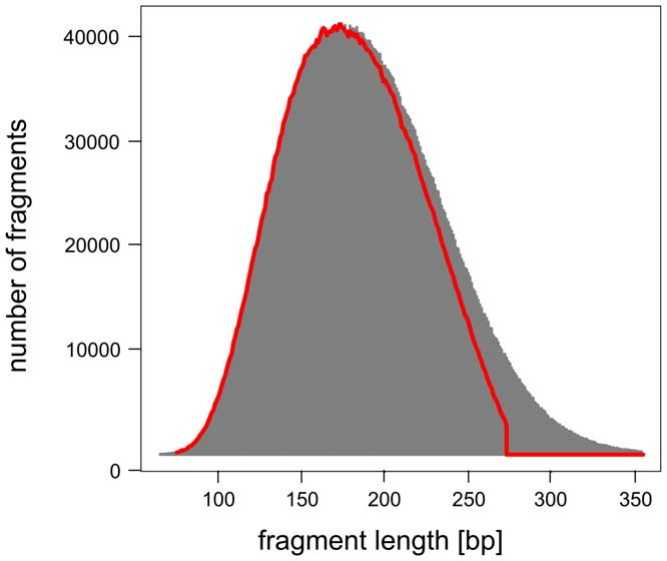
High-confidence alignment yield by insert lengths. Distribution of insert lengths by aligning the original reads to the reference sequence (gray), and lengths of composite reads retained (red).

**Figure 5:**
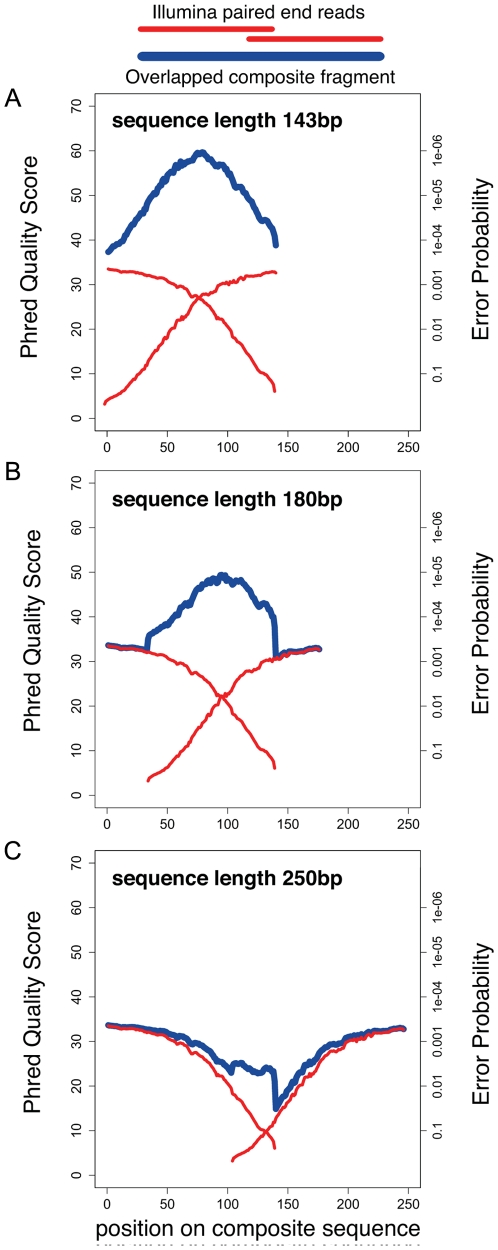
Quality of composite fragments after overlapping. The mean Phred quality (and corresponding error rate) at each position of the read. We show the original Illumina reads and the composite fragments generated by overlapping. **A**) 143 bp, the length of the original Illumina read; **B**) 180 bp, the mean length of overlapped fragment in this library and **C**) 250 bp, roughly the mean length generated by 454-FLX technology.

DNA size selection allowed us to choose an appropriate library insert length with precision, such that mate-pair sequences of each insert overlap in the middle, and can be aligned to produce a longer, more accurate composite read. We used our streamlined dSPRI protocol to produce a paired-end library with an insert size range of 100–300 bp (Figure 1H) for the Illumina GAIIx platform, and generated millions of paired-end reads 143 nucleotides (ntds) in length. We observed that the sequencing error rate remained <1% for 110 ntds in the forward read, and 87 ntds in the reverse read (Figure 3A). To reconstruct each insert's sequence, we developed the SHERA (SHortread Error-Reducing Aligner) software tool, which aligns each mate-pair to produce a composite read with Phred-like quality scores [8]. With each alignment, we report a confidence metric, allowing misaligned reads to be reliably filtered out. We chose a cut-off such that 87% of reads were retained and less than 1% of paired reads were incorrectly aligned. The average composite read length reached 180±40 bp (Figure 4), as expected from the library insert size distribution. Importantly, the composite reads' length can easily be adjusted by modifying dSPRI selection conditions (Figure 1), and reach >200 bp. For nucleotides in the aligned region, SHERA recalculates the error probability of each bp, given the base call data from both reads (Figure 3B). Composite read quality exceeds that of individual short-reads (Figure 3). The shortest composite reads have the best quality scores, still, the average error rate remained <1% across 95% of 250 bp reads (Figure 5). New technologies and sequencing chemistry improvements are likely to further increase ultra high-throughput read lengths, thus extending the achievable length of high-quality composite sequences.

**Figure 6:**
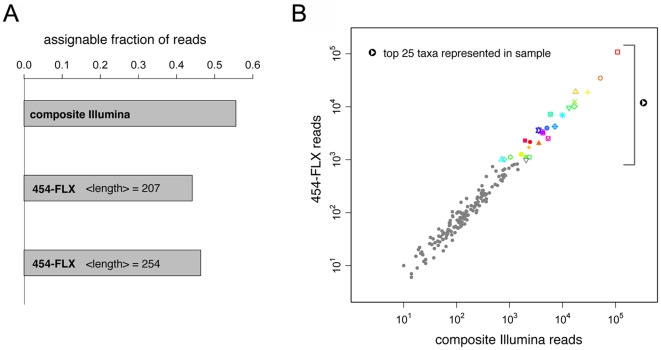
Composite Illumina reads constitute a legitimate alternative to pyrosequencing for metagenomics studies. DNA from a marine metagenomics sample was sequenced with our overlapping mate-pair approach. The composite reads were directly compared to 454-FLX sequences from the exact same sample. **A**) Fraction of reads that could be assigned to a taxon using the composite reads (mean read length 180 bp), the entire 454-FLX dataset (mean read length 207 bp), or longer 454-FLX reads (mean read length 254 bp). **B**) Comparison of taxon assignments using composite reads and 454-FLX pyrosequencing reads. The top 25 represented taxa, with colored symbols next to the bracket, are listed in Table S1.

Short-read technologies have not been widely used for metagenomic analyses because of the difficulty in confidently assigning phylogeny or putative gene function to short sequences, although a strategy using Illumina mate paired libraries to assign taxonomy has recently been evaluated with simulated data [9]. We sought to test if our composite reads could potentially transcend this limitation. To address this question, we sequenced a metagenomic DNA sample from the Hawaii Ocean Time Series [10] using our overlapping paired-end approach and compared the results with those obtained with the Roche 454-FLX technology, the most widely-used platform for these studies. Over 4.8 million high-confidence composite sequences of 180±40 bp were generated from a single sequencing lane of the Illumina GAIIx while 673,673 454-FLX reads of 207±71 bp were already available [10]. Despite the slightly shorter average read length of the composite Illumina reads relative to 454-FLX, the fraction of reads that could be assigned to a taxon was similar in both cases, even when considering longer 454-FLX reads (Figure 6A). The relative abundance of the sequences that could be assigned to each taxon was also consistent between the 454-FLX and composite read datasets (Figure 6B). We have focused our comparison on Illumina and 454-FLX, because they generate similar read lengths, and read length can make a difference in performance for some applications [11]. As the technology landscape evolves our general approach for experimental and computational overlapping of short paired reads will be readily adapted to new platforms and upgrades, and therefore is likely to continue to play a role in providing additional cost-effective sequencing options. Taken together, these results demonstrate that Illumina composite reads produced by SHERA constitute a practical and cost-effective alternative to 454-FLX for metagenome sequencing and analysis while providing significantly deeper sampling at a lower cost.

## Discussion

We have demonstrated that our overlapping mate-pair strategy can substantially increase read length while maintaining high sequence quality, in turn making economical short-read platforms suitable for a range of applications. For example, the performance of phylogenetic classification and annotation of DNA sequences improves with read length, the most significant increase being observed by extending read length from 100 to 200 bp [12]–[13]. By allowing to reach and exceed this latter threshold, our method enables the use of the ubiquitous Illumina platform for metagenomics. Importantly, the dSPRI DNA fragment size selection and the SHERA package for computational read joining can be used with any existing or emerging short-read sequencing platforms capable of producing mate-pairs. Compared to pyrosequencing, the dominant sequencing technology in metagenomics, our composite read strategy already provides a >30-fold increase in sequencing capacity per dollar at similar or higher accuracy (Table 2). We expect that the advances described here will not only unlock short read sequencing for metagenomics, but that a number of other applications including amplicon sequencing, transcriptomics, and de novo assembly, will take advantage of the millions of longer high quality reads produced by this simple and scalable method.

**Table 2:** Cost estimates of sequencing strategies.

Sequencing technology	Composite Illumina	454-FLX
Cost of a run ($)^1^	4,200.00	8,000.00
Number of reads obtained	10,000,000	400,000
Average Read length (bp)	180	250
Reads per $	2381	50
bp per $	428,571	12,500
Cost ratio per read	48	1
Cost ratio per bp	34	1

1 The Illumina sequencing pricing is from the MIT BioMicro center (http://openwetware.org/wiki/BioMicroCenter:Pricing) and the 454-FLX is from http://www.biosystems.usu.edu/core_labs/genomics/454/.

## Materials and Methods

### Double-SPRI (dSPRI) DNA fragment size-selection

High-molecular weight genomic DNA was obtained from a marine environmental sample (Hawaii Ocean Time Series, cruise 186, 75 m depth, [10]) and from laboratory cultures using standard procedures [14], or by amplifying genomes from individually flow-sorted cells as described [15]. DNA samples (50 µL, 2 µg) were sheared using 18–24 cycles of alternating 30 seconds ultasonic bursts and 30 seconds pauses in a 4°C water bath (Bioruptor UCD-200, Diagenode). The resulting DNA molecules were end-repaired and phosphorylated according to manufacturer's recommendations (End-repair kit, Enzymatics or New England Biolabs). The size distribution of the resulting fragments ranged on average from ∼100 to ∼700 bp as judged by Agilent Bioanalyzer DNA-1000 assays.

We optimized a two-step dSPRI protocol [4] for DNA fragment size selection that quantitatively removes fragments above or below the desired size range. In a first separation step, DNA fragments above an upper size threshold are bound by Agencourt AMPure XP SPRI magnetic beads and discarded. In the following second separation step fragments in the desired size range are selectively captured on the beads while all shorter fragments are discarded with the supernatant. The conditions during first and second separation are tunable to enable selective enrichment for other fragment size distributions (Figure 1). In addition, we showed that the size specificity of the dSPRI procedure is stable over a wide range of DNA concentrations (Figure 2). The sequencing libraries prepared for this work were obtained by combining 50 µL of sheared DNA with 40 µL of Agencourt AMPure XP SPRI magnetic beads and incubated at room temperature for 5–7 minutes. The tubes were next placed on a magnet holder (Invitrogen-Dynal) for 2 minutes before transferring the supernatant to a new tube and discarding AMPure XP particles from this first separation step. For the second separation step, 100 µL of fresh AMPure XP beads were added to 85 µL of the supernatant from the previous separation, mixed well, and incubated for 5 minutes before transferring the tube to the magnet holder rack. The supernatant from this second step was discarded and the SPRI particles were washed twice with 0.5 mL of 70% ethanol. The wash steps ensure complete removal of polyethylene glycol (PEG) and salts, but also of any remaining unbound fragments smaller than the desired fragment size distribution. After removing the 70% ethanol from the wash steps, the beads were air dried at room temperature (or at 37°C) for 10–15 minutes. In order to recover the bound DNA, 30 µL of water was added to the beads, and incubated at room temperature for at least 1 minute. The DNA solution was separated from the Agencourt AMPure XP SPRI magnetic beads for 2 min on the magnet holder, and transferred to a new tube. Applying aforementioned conditions for dSPRI led to the specific enrichment of a DNA size-fraction with an average size of 207 bp. Enrichment for other size distribution can easily be achieved by modifying the dSPRI conditions as described in Figure 1.

### Illumina paired-end library construction

Our strategy to create Illumina compatible sequencing libraries uses a blunt-end ligation step of two distinct adapters rather than the ligation of a single Y-shaped adapter [16]. dSPRI-selected DNA fragments were combined with a 10-fold molecular excess for each oligonucleotide adapters (see Table 1 for oligonucleotide sequences). Blunt-end DNA fragments were ligated using a rapid-ligation kit (Enzymatics or New England Biolabs) for 5–10 minutes at room temperature. Adapter excess was subsequently removed using Agencourt AMPure XP SPRI beads according to the manufacturer's recommendations (DNA/bead ratio of 1). A nick-translation step was performed with 2 units of BstI exo^-^ DNA polymerase (New England Biolabs) at 65°C for 25 minutes in a reaction consisting of 20 mM Tris-HCl pH 8.8, 10 mM (NH_4_)_2_SO_4_, 10 mM KCl, 2 mM MgSO_4_, and 0.1% Triton X-100. The resulting DNA fragments were diluted 2 fold with ddH_2_O, and PCR amplified using Phusion Hot Start High-Fidelity DNA polymerase (New England Biolabs), and simultaneously monitored on a Bio-Rad Opticon real-time PCR instrument. Each reaction consisted of 1 µL of diluted nick-translated DNA, 0.5 units of Phusion Hot Start High-Fidelity DNA polymerase, 1X Phusion HF buffer, 0.4 µM of each IGA-PCR-PE-F and IGA-PCR-PE-R primers [described in Table 1], 200 µM dNTPs as well as 0.25X SYBR green I. The initial denaturation step was at 98°C for 1 minute, followed by successive cycles of 98°C for 15 seconds, 65°C for 10 seconds and 72°C for 15 seconds. The reactions were stopped in the late logarithmic amplification phase, and the DNA from the corresponding samples were pooled. The resulting libraries were composed of fragments with distinct adapters at each extremities due to PCR suppression effect [17]–[19]. The amplified libraries were subjected to an additional round of AMPure XP SPRI beads purification, with a DNA/beads ratio of 1, to remove residual primers and adapter dimers.

In order to achieve optimal cluster densities for sequencing on the Illumina GAII platform, samples were quantified using two methods. The samples were first analyzed using the High-Sensitivity DNA Kit for the Bioanalyzer (Agilent Technologies) to detect any primer-dimers and to determine the average molecular weight of each library. The samples were next quantified by real-time PCR on a LightCycler II 480 (Roche), using the LightCycler 480 SYBR Green I Master mix (Roche), appropriate primers (Table 1), and serially diluted PhiX 335 bp control library (Illumina) as a standard (quantified with the Agilent Bioanalyzer; we observed that the Illumina PhiX 335 bp concentration vary from lot to lot, and typical concentrations were found to range from 11–15 nM). The phiX standard curve was required to have an r^2^ value of >0.98 or the run was repeated. This step both confirmed the concentration of the libraries as well as confirmed the presence of proper primers for Illumina sequencing.

### Illumina paired-end sequencing

Illumina libraries were clustered for sequencing immediately following sample quantification according to manufacturers recomendations (Illumina). Following clustering, the samples were loaded on to an Illumina GAIIx sequencer. Sequencing reagents for each read were pooled prior to loading. Data from the Illumina GAIIx was analyzed using the Illumina pipeline 1.4.0 to generate fastq files.

### Mate-Read Overlapper Algorithm

In order to align overlapping mate reads and produce accurate composite fragments, we developed the SHERA software pacakge, a collection of computer code written in the PERL programming language. The algorithm for aligning paired reads and computing quality scores was designed to be broadly applicable, independent of the sequencing platform. The source code is freely available at http://almlab.mit.edu/ALM/Software/Software.html. In brief, SHERA takes overlapping short reads as input, finds the best alignment, produces a composite read, calculates new quality scores for the overlapping bases, and scores the alignment confidence. A detailed description of each of these steps is provided below. We also describe our procedure for evaluating false alignment rates on both real and simulated reads.

### Finding the best alignment

For each mate-pair, the algorithm scores all possible ungapped alignments (this includes alignments shorter than the read length, which are produced when a fragment is fully sequenced resulting in and additional synthesis reactions proceed past its 5-prime end, base-pairing with the Illumina adapters.) Each alignment is scored by summing over all matches and subtracting a penalty for each mismatch. For the alignment with the best score, the algorithm constructs the consensus sequence in this overlapping region by reporting the nucleotide with the higher quality value, or the informative base call if one nucleotide is 'N'. A Phred quality score is calculated for each consensus base (see below). The algorithm output includes sequence and quality of the composite read generated from each mate pair, as well as an alignment confidence score that enables filtering out probable mis-alignments.

### Alignment confidence metric

Alignment confidence was quantified in order to identify composite reads constructed from mis-alignments. Due to the relatively short alignment lengths with non-uniform base composition and quality, we did not model the statistical significance of an alignment analytically, but instead chose an empirical metric. Because mis-alignments of similar length have similar alignment scores (within noise), we use seven alignments of similar length of the same mate-pair to calculate a baseline, and divide by the range of the scores to estimate the alignment's significance. Alignments of all confidence levels are reported; we leave selection of this threshold to the user to achieve the sensitivity/specificity required of their downstream application.

### Calculation of a Phred Quality Score for the overlapped region

SHERA reports a Phred-scaled error probability for each base in the composite read. For overlapped bases, this is the posterior probability that the consensus base is wrong, given the base calls at that position in the alignment and their respective quality scores. Calculating this probability required making a few assumptions and approximations:

1. Given a basecall was in error, there is a uniform probability of reporting one of the other three bases.
2. The probability of correct alignment was approximated to be 1. (A decent approximation given true-positive rates of >99% in our quality filtered alignments, see Counting False Alignments below).
3. The basecalls of the forward and reverse read are independent observations, given the true base in the insert.
4. The prior probability of a base being present in an insert in this flowcell can be estimated from the low-error portion of the reads or approximated as uniform at the user's discretion (In our case, we used empirical observations to estimate %GC in single-genome samples, and a uniform distribution for the metagenomic sample.)

We first confirmed that the posterior error probability for the overlapped region was correct by counting the true error rate in composite reads constructed from simulated reads. Next, using real mate-reads as input, we confirmed that our Phred quality scores up to 32 correspond to the correct error rate as calculated by comparing to the reference. Above 32, the error rate does not improve (for neither the overlapped bases nor the original Illumina bases). This is due to errors in the reference and/or a very small percentage of Illumina reads with insertions or deletions.

### Counting False Alignments

Minimizing the number of false alignments is an important step for some applications such as de novo assembly. Our goal was to find a threshold for alignment confidence that would allow no more than 1% misalignments to remain in our subset of composite reads (based on previous experience with de novo assembly of libraries containing hybrid reads). We used several approaches to assess our alignment accuracy and found similar results.

First, we used the read-simulator from the MAQ package [20] to produce simulated reads that mimicked the error profiles observed in the quality values of our Illumina data as well as the distribution of overlap lengths. In the case of simulated reads, the true distance between mate pairs is known exactly and the reference is perfect. After filtering (confidence metric ≥0.5) we retain 89.7% of the composite reads, 0.5% of which were constructed from mis-alignments. This strategy is useful when no reference is available, or as a test case on new platforms.

**Figure 7:**
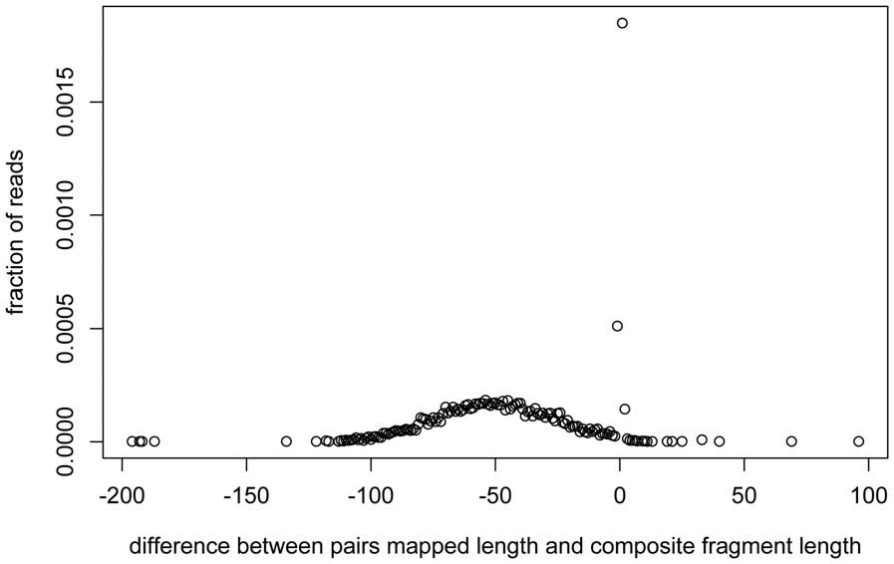
Short insertions and deletions in low-confidence composite Illumina reads. Mate-reads from the control lane (PhiX174 bacteriophage genome) were used to assess false alignments introduced by the SHERA pipeline. After constructing the composite sequences and filtering, we plotted the difference in length (if any) between a composite fragment and its insert length as predicted by MAQ by mapping the original mate-pairs to the reference.This histogram is a sum of two distributions, the overlapper software's misalignments (a broad gaussian) and a sharp peak of small (1–2 bp) indels. We used a simple and conservative linear model to remove the indels and infer the number of misalignments.

Second, we used mate-reads from the PhiX control lane sequenced during our Illumina sequencing run. This DNA library is prepared from the PhiX174 bacteriophage genome for purposes of quality control, and we found that it had and insert length 200±21 bp (estimated by mapping to the reference with MAQ). These mapped mate-pairs defined the “true” insert length of each sequenced fragment. After constructing the composite sequences and filtering, we plotted the difference in length between a composite fragment and its insert length as predicted by MAQ (Figure 7). This histogram is a sum of two distributions, the overlapper software's misalignments (a broad gaussian) and a sharp peak of small (1–2 bp) indels. We examined gapped alignments to confirm this was the case and also found that many of the insertion or deletions occur in homopolymer runs. We used a simple linear model to infer the number of misalignments; this yielded a false positive rate of 1.0% for PhiX174.

Finally, for Figure 3C, we assessed alignment yield and accuracy on mate-pair reads sequenced from a dSPRI library. This library was generated from a single Prochlorococcus cell (as described [15]), for which a draft copy of the genome (N50 = ∼7 Kb) was constructed independently by de novo assembly of 454-Titanium reads from the same DNA sample. We mapped the Illumina mate-paired reads to these 454-Titanium contigs to define the “true” insert length of each sequenced insert. A small fraction of mate-pairs (<5%) were unmapped or mapped to different contigs; these were excluded from further analysis. As in the case of PhiX174, there were a number of small indels evident in the comparison between reads and reference which were excluded from our statistic. We estimate that 0.5% of our high confidence set of composite reads were mis-alignments introduced by SHERA.

### Comparison of 454-FLX and overlapped Illumina reads for metagenomic applications

To evaluate the suitability of overlapped Illumina for metagenomic analysis, we made a direct comparison to the Roche 454-FLX platform, currently the most commonly used sequencing platform for this application. We used sequencing data from the exact same marine metagenomic sample [10]. We asked whether reads from the two platforms were equally able to identify significant protein homology with known proteins for the purposes of taxonomic classification.

We obtained 6,339,825 mate-paired Illumina reads of length 143 ntds each with insert lengths shown in Figure 3. We joined the Illumina mate-pair reads with our algorithm and filtered the composite reads by alignment confidence, resulting in 4,830,552 high-confidence composite reads of length 180±40 bp. We analyzed both high-confidence and low-confidence sets of composite reads as described below. We also had access to 673,673 454-FLX reads of length 207±71 ntds available for comparison [10]. We blasted all 454-FLX reads and composite Illumina reads against the NCBI protein database nr [21] using translated nucleotide queries (blastx parameters -b 100 -v 100 -e 30 -Q 11 -F “m S”), and used the popular metagenomics software MEGAN [22] for downstream analysis. We compared the fraction of unassignable reads (due to lack of significant blast hit in the database) across four subsets of sequence data from the same sample, which were chosen to compare sequencing platforms and the effect of read length: high-confidence composite Illumina reads, low-confidence composite Illumina reads, a representative sample of our 454 reads (mean length 207 bp), a longer subset of our 454 reads (mean length 254 bp). For comparison purposes we used random samples of 50,000 reads each. We used the MEGAN parameters recommended by the authors [22] as suitable for comparing reads of different lengths (minscore = 35.0, minscorebylength = 0.35, toppercent = 10, minsupport = 1; the low minsupport value was chosen because some of the data subsets tested were necessarily small. We also tested the alternative parameter set with similar results: minscore = 40.0, toppercent = 10, minsupport = 5). In Figure 6A we show the fraction of DNA fragments for which MEGAN was able to assign to a known genome or taxon via significant protein homology. We show that composite Illumina reads from high-confidence alignments perform at least as well as reads obtained from 454-FLX. We also find that composite reads produced by alignments we designated as “low-confidence” have a very high fraction of unassignable reads, further proof of the ability of our algorithm to identify misalignments. Finally, we show results for 454-FLX reads of mean length 254 bp (range: 230–270 bp), more typical of datasets produced by the 454-FLX platform. The assignable read fraction is comparable.

Next we asked if the taxonomic classification was consistent across platforms. We used MEGAN [22] to classify all of the 454-FLX reads and an equal number of composite Illumina reads. Figure 6B shows the taxonomic classification is relatively invariant with respect to the sequencing technology chosen. We performed this analysis with two reasonable parameters sets (minscore = 35.0, minscorebylength = 0.35, toppercent = 10, minsupport = 5 OR minscore = 40.0, toppercent = 10,minsupport = 5) and observed equally good agreement in taxonomic classification across platforms.

## Supporting Information

Table S1 Top 25 taxa identified in the HOT186 75 meters depth metagenomics sample.
(0.80 MB PDF)
