# Supplementary material for: Unlocking Short Read Sequencing for Metagenomics
Source: PLoS One. 2010 Jul 28;5(7):e11840. doi: 10.1371/journal.pone.0011840 (PMC2911387; doi:10.1371/journal.pone.0011840)
Supplement: Table S1 — Top 25 taxa identified in the HOT186 75 meters depth metagenomics sample. (0.80 MB PDF) [file pone.0011840.s001.pdf]

- Prochlorococcus marinus
- Bacteria
- Candidatus Pelagibacter
- cellular organisms
- root
- Proteobacteria
- Cyanobacteria
- Candidatus Pelagibacter sp. HTCC7211
- Alphaproteobacteria
- Gammaproteobacteria
- Bacteroidetes
- SAR11 cluster
- Psychroflexus torquis ATCC 700755
- Prochlorococcus phage P-SSM2
- Alteromonas macleodii
- Candidatus Pelagibacter ubique
- Not assigned
- Rhodobacterales
- Viruses
- Flavobacteria
- Prochlorococcus phage P-SSP7
- Candidatus Pelagibacter ubique HTCC1062
- Alteromonas macleodii ATCC 27126
- Prochlorococcus marinus str. AS9601
- Prochlorococcus marinus str. MIT 9312
